# Drug-eluting intraocular lens with sustained bromfenac release for conquering posterior capsular opacification

**DOI:** 10.1016/j.bioactmat.2021.07.015

**Published:** 2021-07-23

**Authors:** Xiaobo Zhang, Kairan Lai, Su Li, Jing Wang, Jiayong Li, Wei Wang, Shuang Ni, Bing Lu, Andrzej Grzybowski, Jian Ji, Haijie Han, Ke Yao

**Affiliations:** a Eye Center, Second Affiliated Hospital, School of Medicine, Zhejiang University, Hangzhou, PR China; b Zhejiang Provincial Key Laboratory of Ophthalmology, Hangzhou, PR China; c Department of Ophthalmology, University of Warmia and Mazury, 60-554 Olsztyn, Poland; d Institute for Research in Ophthalmology, Gorczyczewskiego 2/3, 61-553 Poznan, Poland; e MOE Key Laboratory of Macromolecule Synthesis and Functionalization of Ministry of Education, Department of Polymer Science and Engineering, Zhejiang University, Hangzhou, PR China

**Keywords:** Posterior capsular opacification, Drug-eluting IOLs, Bromfenac, ERK pathway, Transcription factor Snail

## Abstract

Cataract is the leading cause of visual impairment, and posterior capsular opacification (PCO) is the most common long-term complication of modern cataract surgery, which can cause severe visual impairment after surgery. The proliferation, migration, and epithelial-mesenchymal transition (EMT) of residual lens epithelial cells (LECs) stimulated by growth factors and cytokines, are the key pathological mechanisms involved in the development of PCO. This study demonstrated that non-steroidal anti-inflammatory drug (NSAID), bromfenac, was capable of effectively inhibiting cell migration, overexpression of EMT markers, such as fibronectin (FN), matrix metalloproteinase 2 (MMP2), α-smooth muscle actin (α-SMA), and transcription factor Snail, and extracellular signal-regulated kinase (ERK)/glycogen synthase kinase-3β (GSK-3β) signaling induced by transforming growth factor-β2 (TGF-β2) *in vitro*. The inhibitory effect of bromfenac on TGF-β2-induced EMT was also verified on a primary lens epithelial cell model using human anterior capsules. Furthermore, based on ultrasonic spray technology, we developed a drug-eluting intraocular lens (IOL) using poly (lactic-co-glycolic acid) (PLGA) with sustained bromfenac release ability for the prevention of PCO development. In the rabbit models of cataract surgery, bromfenac-eluting IOL exhibited remarkable PCO prevention and inflammation suppression effects with excellent biocompatibility. In conclusion, bromfenac can inhibit TGF-β2-induced cell migration and the EMT of LECs *via* ERK/GSK-3β/Snail signaling. The present study offers a novel approach for preventing PCO through PLGA-based drug sustained-release IOLs.

## Highlights

• Bromfenac inhibited TGF-β2-induced migration and EMT of LECs through ERK/GSK-3β/Snail signaling.
• Drug-eluting IOLs with sustained bromfenac release were developed based on ultrasonic spray technology.
• Bromfenac-eluting IOLs exhibited remarkable PCO prevention and inflammation suppression effects *in vivo.*
• Bromfenac-eluting IOLs hold great potential for clinical application of PCO prevention.

## Graphical abstract

TGF-β2 promoted migration and EMT of lens epithelial cells (LECs) through activation of ERK/GSK-3β/Snail signaling, which could be suppressed by bromfenac. Novel bromfenac-based drug-eluting IOLs exhibited excellent PCO prevention *in vivo*.

**Figure:**
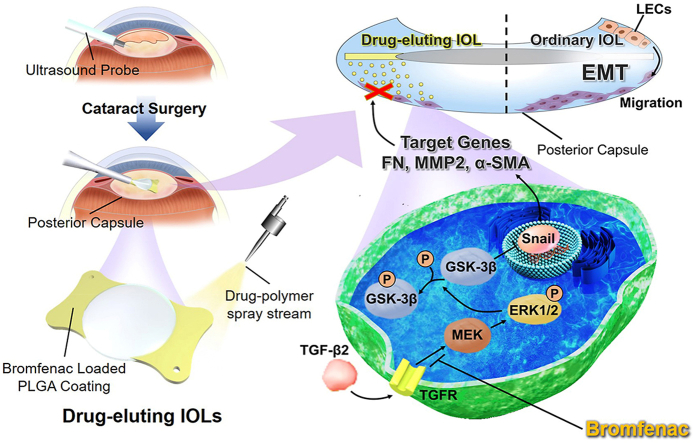

## 1 Introduction

Cataract is the leading cause of visual impairment in the middle- and low-income countries [1], and cataract surgery is the only effective treatment [2]. However, the postoperative visual outcomes of patients are limited by posterior capsular opacification (PCO), the most common long-term complication of modern cataract surgery. The incidence of PCO is reported to be 20%–60% in adults [3] and can even reach 95% or more in young people since the cells of adolescents and children have greater regenerative and proliferative capacities [4]. Improvements in surgical techniques and the innovation of intraocular lens (IOL) design and materials were adopted to reduce the incidence of PCO, but the effect is not significant, especially in children [5,6]. PCO causes severe visual impairment after surgery, and the primary treatment for PCO in clinic is Nd: YAG laser posterior capsulotomy. However, it also has many risks, such as corneal edema, uveitis, anterior chamber hemorrhage, cystoid macular edema, elevated intraocular pressure, and even malignant glaucoma [7,8]. Therefore, it is vital to understand the formation and development mechanisms of PCO, as well as explore prevention strategies.

PCO is attributed to the fact that residual lens epithelial cells (LECs) proliferate and migrate from their original position to gradually cover the posterior capsule, stimulated by several growth factors and cytokines after cataract surgery [9,10]. The epithelial-mesenchymal transition (EMT) of LECs is the central pathogenesis of PCO, a wound-healing response, and the morphology, polarity, and migration capabilities of LECs change during EMT development [11,12]. In the wound-healing response process following cataract surgery, residual LECs undergo cytoskeleton remodeling and fibrosis, whose characteristics are irregular deposition of extracellular matrix proteins, including collagen, fibronectin (FN), matrix metalloproteinases (MMPs), and α-smooth muscle actin (α-SMA) [13,14]. Bromfenac is a non-steroidal anti-inflammatory drug (NSAID) commonly used in ophthalmology and mainly exerts its anti-inflammatory effect by inhibiting COX-2 with low cytotoxicity [15]. Brookshire et al. [16] found that bromfenac was more effective in inhibiting the occurrence of PCO in experimental dogs than prednisolone acetate. However, whether bromfenac can effectively inhibit EMT of LECs and the potential mechanism of PCO inhibition by the NSAID bromfenac is still unclear.

Transforming growth factor-beta (TGF-β), as an essential growth factor, is highly involved in the EMT and pathological fibrosis of LECs. In particular, the presence of TGF-β2 in the aqueous humor is of great significance for the development of PCO. Several signaling pathways are involved in this process. Aomatsu et al. [17] used TGF-β to stimulate human corneal epithelial cells (HCECs) and found an over-expression of vimentin (VIM), FN, and transcription factors, including Snail and Slug. But the specific inhibitors of mitogen-activated protein kinase/extracellular signal-regulated kinase kinase (MAPK/ERK kinase, MEK) could prevent this upregulation process to some degree, indicating that the extracellular signal-regulated kinase (ERK) pathway plays a vital role in the EMT process.

The ERK signaling pathway plays a vital role in cell proliferation, differentiation, migration, apoptosis, and the EMT promotion of epithelial cells [18,19]. Liu et al. [20] found that, in LECs, the MEK-specific inhibitor could almost wholly inhibit the activation of ERK1/2 triggered by TGF-β2. In contrast, the inhibitor of the TGF-β/Smad2/3 pathway did not affect the activation of ERK1/2, indicating that the activation effect of the ERK1/2 pathway triggered by TGF-β2 on regulating EMT was not controlled by the TGF-β/Smad pathway. Glycogen synthase kinase-3β (GSK-3β), a serine/threonine kinase widely found in mammalian cells, is closely related to cell proliferation, apoptosis, migration, and EMT [[21], [22], [23]] and can phosphorylate, ubiquitinate, or degrade transcription factors, including Snail and Slug to maintain epithelial phenotypes [24,25]. Therefore, we infer that when GSK-3β is phosphorylated and inactivated, the degradation of a range of downstream transcription factors, such as Snail and Slug, is limited, which can promote the development of EMT.

**Scheme 1:**
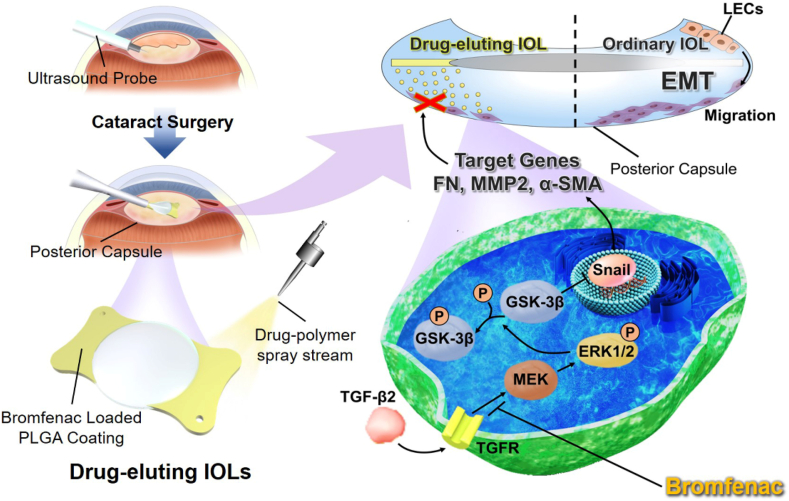
**The schematic illustration ofthebromfenac-eluting intraocular lens as a novel approach for the prevention of PCO.** The ERK signaling can be activated by TGF-β2, which leads to the inactivation of GSK-3β by phosphorylation. GSK-3β can phosphorylate and degrade Snail, which plays a role as a transcription factor to regulate FN, MMP2, and α-SMA to promote cell migration and EMT. Bromfenac can efficiently suppress the phosphorylation of ERK1/2 induced by TGF-β2. We developed bromfenac-eluting intraocular lens (IOL) spray-coated with PLGA carrying bromfenac on the plate haptics, which was designed as a drug delivery system for the prevention of PCO development.

Recently, IOL modification is considered as a new potential strategy for PCO prevention [6,[26], [27], [28]]. However, it is still challenging to maintain the refractive properties of IOLs and also avoid possible damage to intraocular tissue. The present study firstly verified the role of the ERK signaling pathway in the TGF-β2-induced EMT of LECs and elucidated the potential mechanism by which bromfenac suppressed its progress. Due to the corneal absorption barrier, the bioavailability of drugs in intraocular tissues through eye drops is relatively low, with less than 3% [[29], [30], [31]]. Herein, we subsequently designed and prepared a novel drug-eluting IOL based on ultrasonic spray technology using poly (lactic-co-glycolic acid) (PLGA) which has been used as drug carriers in ophthalmic implantation (Ozurdex®) to achieve sustained intraocular release of bromfenac as shown in Scheme 1 [32,33]. The PLGA with bromfenac was precisely sprayed onto the plate haptics of IOL, ensuring the smoothness and transparency of the IOL's optics, which was beneficial for clinical translation. The PCO prevention effect of the novel drug-eluting IOL with sustained drug release property was investigated in a rabbit PCO model. Together, rely on the drug mechanism research and novel IOLs design, we provide a promising approach for the prevention of PCO.

## 2 Materials and methods

### 2.1 Cell culture and reagents

A human lens epithelial cell line (HLEC-B3) was obtained from the American type culture collection (ATCC, Manassas, VA, US) and grown in Dulbecco's Modified Eagle Medium—Nutrient Mixture F-12 (DMEM/F12; Gibco, Grand Island, NY, US), containing 10% fetal bovine serum (FBS; Gibco) at 37 °C in a 5% CO_2_ humidified atmosphere.

PLGA (lactide: glycolide 75:25, Mw: 40,000-75,000) was provided by Sigma Chemical Co. (St. Louis, MO, USA). Bromfenac and 0.1% bromfenac ophthalmic solution were obtained from Senju Pharmaceutical (Osaka, Japan). Recombinant human TGF-β2 and U0126 were purchased from Cell Signaling Technology Inc. (CST, Danvers, MA, US), and CHIR-99021 was purchased from Selleck Chemicals (Houston, TX, US).

### 2.2 Construction of bromfenac-eluting IOLs

The 5% PLGA solution in ethyl acetate and bromfenac solution in DMF was prepared and then co-sprayed onto the plate haptics of custom-made hydrophobic acrylic IOLs (Wuxi Vision Pro Ltd., Wuxi, China) with an ultrasonic spraying system (Ruidu Photoelectric Technology Co. Ltd, Shanghai, China). The coating was only sprayed onto the plate haptics of IOLs, and the optics of the IOLs remained smooth and transparent to ensure the clarity of IOLs. Each IOL was spray-coated with 100 μg of bromfenac onto the plate haptics. Finally, three types of IOLs were involved in this study, which are: control-IOLs = IOLs were left uncoated, PLGA-IOLs = IOLs were spray-coated with PLGA; bromfenac-PLGA-IOLs = IOLs were spray-coated with bromfenac.

### 2.3 Surface characterization and drug-release kinetics of bromfenac-eluting IOLs

Fourier transform infrared spectroscopy in attenuated total reflection mode (ATR-FTIR) and X-ray photoelectron spectroscopy (XPS) were applied to characterize the surface chemical compositions of the coating on IOLs. ATR-FTIR data were obtained with a BRUKER TENSOR Ⅱ spectrometer (Germany). XPS data were acquired with a Thermo Scientific K-Alpha + spectrometer (US) equipped with a monochromatic Al Kα X-ray source (1486.6 eV). The water contact angle was applied to characterize the surface wettability of the coating on IOLs with a Kruss DSA 100 system (Germany). Five μL of distilled water was applied with a syringe needle on the coating for each measurement. An ultraviolet–visible spectrometer (UV–vis; Thermo Scientific Evolution 300, US) was involved to detect the transmittance of the optical center of IOLs. Scanning electron microscopy (SEM; Hitachi S4800, Japan) was performed to observe the morphology of the coating on IOLs.

The release of bromfenac from drug-eluting IOLs was observed using a UV–vis spectrometer with a PBS medium. First, the standard curve of bromfenac was acquired by establishing a relationship between the drug concentration and corresponding absorbance value at a constant characteristic wavelength peak (268 nm) using the UV–vis spectrometer. Then, each bromfenac-eluting IOL was immersed in 2 mL PBS in a prepared tube, and the prepared tube was then placed in a thermostated incubator and constantly shaken (100 rpm) at 37 °C. At pre-determined time points, 1 mL of media was removed and replaced with a fresh medium [34]. The absorbance value of released bromfenac was measured and used to calculate the corresponding drug concentration based on the standard curve. Finally, through dynamic detection and repeated experiments, the release curve of bromfenac was obtained.

### 2.4 Total RNA extraction and quantitative real-time polymerase chain reaction (qPCR)

The total RNA of the cells was extracted using TRIzol™ reagent (Invitrogen, Carlsbad, CA, US) according to the manufacturer's instructions. Reverse transcription reactions were performed with quantified total RNA using a PrimeScript™ RT reagent kit (Takara, Shiga, Japan). For the quantitative analysis of mRNA expression, an SYBR Premix Ex Taq™ kit (Takara, Shiga, Japan) was used for real-time qPCR on a 7500 Fast Real-Time PCR System (ABI, CA, US) [35]. The primers are listed in Table 1. The expression levels of the target genes were normalized to GAPDH levels and calculated using the delta-delta Ct method.

**Table 1:** Primer sequences.

Gene	Sequences		Product length(bp)
Snail	Forward	5′-CCCCAATCGGAAGCCTAACT-3′	62
	Reverse	5′-GCTGGAAGGTAAACTCTGGATTAGA-3′	
Fibronectin	Forward	5′-TCGAGGAGGAAATTCCAATG-3′	267
	Reverse	5′-ACACACGTGCACCTCATCAT-3′	
MMP2	Forward	5′-GGCCCTGTCACTCCTGAGAT-3′	474
	Reverse	5′-GGCATCCAGGTTATCGGGGA-3′	
α-SMA	Forward	5′-ATAACATCAAGCCCAAATC-3′	245
	Reverse	5′-ACTTCCCAAAGCATCAGC-3′	
GAPDH	Forward	5′-TGCACCACCAACTGCTTAGC-3′	87
	Reverse	5′-GGCATGGACTGTGGTCATGAG-3′	

### 2.5 siRNA transfection

siRNA transfection was performed using Lipofectamine 2000 reagent (Invitrogen, Carlsbad, CA, US) and a reduced serum medium (Opti-MEM, Gibco, Grand Island, NY, US) according to the manufacturer's instructions. The sequences of the siRNAs employed in this study were: Snail (sense, 5′-GGA CAA AGG CUG ACA GAC UTT-3’; anti-sense, 5′-AGU CUG UCA GCC UUU GUC CTT-3′), and negative control (NC) siRNA (sense, 5′-UUC UCC GAA CGU GUC ACG UTT-3’; anti-sense, 5′-ACG UGA CAC GUU CGG AGA ATT-3′). The Snail siRNA and NC siRNA were synthesized by GenePharma Co. (Shanghai, China). In this experiment, the cells were divided into six groups as follows: 1) NC siRNA group = cells were treated with NC siRNA transfection only; 2) Snail siRNA group = cells were treated with Snail siRNA transfection only; 3) NC siRNA + TGF-β2 group = cells were treated with NC siRNA transfection and induced with TGF-β2; 4) Snail siRNA + TGF-β2 group = cells were treated with Snail siRNA transfection and induced with TGF-β2; 5) NC siRNA + TGF-β2+ bromfenac group = cells were treated with NC siRNA transfection and bromfenac and induced with TGF-β2; 6) Snail siRNA + TGF-β2+ bromfenac group = cells were treated with Snail siRNA transfection and bromfenac and induced with TGF-β2.

### 2.6 Western blot analysis

After being washed twice with ice-cold PBS, the cells were harvested and lysed with lysis buffer (Sangon, Shanghai, China) containing a protease inhibitor cocktail (Sangon, Shanghai, China). The protein concentration was assessed using the bicinchoninic acid Protein Assay Kit (Thermo Scientific, Grand Island, NY, US). Equal amounts of protein were separated by 10%–15% sodium dodecyl sulfate-polyacrylamide gel electrophoresis (SDS-PAGE, and then transferred to polyvinylidene fluoride (PVDF) membranes (Millipore, Billerica, MA, US), blocked in Tris-buffered saline with Tween® 20 (TBST) containing 5% bovine serum albumin (BSA). The PVDF membranes were incubated overnight at 4 °C with the following primary antibodies: Snail (1:2,000, CST, US); p-Smad2/3 (1:2,000, CST, US); p-ERK1/2 (1:1,000, CST, US); GSK-3β (1:1,000, CST, US); p-GSK-3β (1:1,000, CST, US); MMP2 (1:1,000, Proteintech, Rosemont, IL, US); FN (1:2,000, Proteintech, Rosemont, IL, US); α-SMA (1: 5000, Abcam, Cambridge, UK); GAPDH (1:1,000, CST, US); and β-actin (1:1,000, CST, US). The membranes were then washed three times with TBST and incubated with horseradish peroxidase-conjugated secondary antibody (1:5,000, CST, US) for 1 h at room temperature. The immunoreactive bands were developed using ECL-chemiluminescence (Millipore, Billerica, MA, US) and visualized with a ChemiDoc XRS System (Bio-Rad Laboratories, CA, US). The resulting protein bands were analyzed using Image J software (NIH, Bethesda, MD, US). GAPDH and β-actin were used as the internal control.

### 2.7 Immunofluorescence assay

The HLECs were seeded on sterilized coverslips and cultured with DMEM/F12 containing 10% FBS in 24-well plates. After treatment with the indicated reagents, the cells were fixed with 4% paraformaldehyde for 20 min at room temperature, and then permeabilized in 0.3% Triton X-100 (Sigma-Aldrich, St. Louis, MO, US) for 15 min at 4 °C and blocked with goat serum (Boster, Wuhan, China) for 2 h at room temperature. After that, the cells were incubated with anti-rabbit MMP2 antibody (1:200, Abcam, Cambridge, UK), anti-rabbit FN antibody (1:200, Abcam, Cambridge, UK), or anti-rabbit α-SMA antibody (1:200, Abcam, Cambridge, UK) overnight at 4 °C, and then washed three times with PBS, incubated with Alexa Fluor 488-conjugated secondary antibody (1:1,000, CST, Danvers, MA, US) for 2 h at room temperature. To localize the cells and make the staining of target proteins more convenient to observe, the nuclei of the cells were stained with 4′,6′-diamidino-2-phenylindole (DAPI, Sigma-Aldrich, St Louis, MO, US) for 15 min. The images were captured with a fluorescence microscope (Olympus, Tokyo, Japan).

### 2.8 Wound-healing assay

3 × 10^5^ HLECs were seeded in 6-well plates and cultured for 24 h. The cells were divided into four groups as follows: 1) no treatment (control group); 2) treated with 10 ng/mL TGF-β2 only; 3) treated with 10 ng/mL TGF-β2 and 40 μg/mL bromfenac; 4) treated with 10 ng/mL TGF-β2 and 80 μg/mL bromfenac. Briefly, after pre-treatment with bromfenac for 1 h, the cells were scratched to generate wounds using a sterile pipette tip. The cells were incubated in serum-free DMEM/F12 for 24 h and then stimulated with TGF-β2. Images were captured at 0 and 48 h after scratching by microscopy to evaluate cell migration. The wound area was measured using Toupview software (ToupTek Photonics, Hangzhou, China): more than three random fields were selected for assessment, and the wound area at the 0 h time point was used as a reference.

### 2.9 Human anterior capsule culturing and analysis

Continuous curvilinear capsulorhexis is an important step in cataract surgery. Usually, the anterior capsules torn off during surgery will be discarded. In this study, twenty-one different human anterior capsule samples were obtained from patients undergoing cataract surgery. This study was undertaken following the Declaration of Helsinki and approved by the Institutional Review Board of our institute, and written informed consent was obtained from each patient. The anterior capsule samples were cultured in DMEM/F12 containing 10% FBS, 100 U/mL penicillin, and 100 g/mL streptomycin (Gibco Life Technologies, US). After 24 h, the culture medium was replaced by DMEM/F12 containing 1% FBS, with or without bromfenac at a concentration of 80 μg/mL, and treated with 10 ng/mL TGF-β2. After incubation for 48 h, the anterior capsules were taken out of the culture medium and washed three times with PBS. Subsequently, the anterior capsules were fixed with 4% paraformaldehyde for 20 min at room temperature and washed three times, then permeabilized in 0.5% Triton X-100 for 15 min 4 °C. After being washed a further three times, the anterior capsules were blocked with PBS containing 1% BSA for 30 min at room temperature. Thereafter, the anterior capsules were incubated with Alexa Fluor 555-Phalloidin (1:100, Invitrogen, Carlsbad, CA, US) for 30 min in darkness at room temperature. After a further three washes, the anterior capsules were flat-mounted on microscope slides and visualized using an inversion fluorescence microscope (Leica, Wetzlar, Germany).

### 2.10 Cataract surgery and IOL implantation *in vivo*

All animal experiments were performed in accordance with the ARVO Statement for the Use of Animals in Ophthalmic and Vision Research the Guidelines for the Care and Use of Laboratory Animals (NIH publication 85-23, revised 1985). Forty female Japanese white rabbits (12-weeks-old, bodyweight 2.0–2.5 kg) were used in the experiment, which was approved by the Laboratory Animal Ethics Committee of Zhejiang University. The experimental rabbits were divided into four groups (n = 10) randomly as follows: 1) control-IOL (Ctrl-IOL) group = implanted control-IOLs; 2) PLGA-IOL group = implanted PLGA-IOLs; 3) control-IOLs with bromfenac eye drops (Brom drops) group = implanted control-IOLs and received 0.1% bromfenac eye drops four times daily for four weeks after surgery; 4) bromfenac-PLGA-IOL (Brom-PLGA-IOL) group = implanted bromfenac-PLGA-IOLs. All animals underwent phacoemulsification cataract extraction and intraocular lens implantation in the right eye only. All surgeries were performed by the same surgeon (Dr. Wei Wang) while the animals were anesthetized with an IV injection of 1 mL/kg of body weight with 3% pentobarbital sodium. Pupillary dilation was induced by one drop of 0.5% tropicamide (Santen, Japan), administered three times at intervals of 15 min. Pranoprofen (Senju Pharmaceutical Co. Ltd., Japan) and 1% Atropine Ointment (Sinqi Pharmaceutical Co., Ltd., Shenyang, China) were applied three times daily before surgery. Two clear corneal incisions (CCIs), including a 3.0 mm primary incision and a 1.0 mm secondary incision, were created with keratomes and, after phacoemulsification had been performed with a Stellaris system (Bausch & Lomb, USA), the different IOLs were implanted into the eyes of the different groups, respectively.

The postoperative regimen included topical administration of dexamethasone-tobramycin (Alcon Laboratories, Inc., Belgium) and pranoprofen four times daily for two weeks. The experimental eyes were examined with a slit lamp photography system (66 Vision-Tech Co. Ltd., Suzhou, China) on the third, seventh, fourteenth, and twenty-eighth days after surgery. PCO development was evaluated using retro illumination photographs in accordance with the following criteria [36]: 0 = none, no PCO; 1 = slight, PCO not reaching the edge of the optic; 2 = moderate, PCO reaching the edge; 3 = pronounced, PCO beyond the edge, but the visual axis is clear; 4 = severe, PCO covers the visual axis.

The rabbits were sacrificed with an injection of overdose anesthesia at the end of the experiment. The right eye tissues of the rabbits were enucleated to evaluate PCO development from the posterior aspect (Miyake-Apple view) and used for histological examination. The ocular tissues, including the posterior capsules, corneas, irises, and retinas, were assessed using hematoxylin and eosin (H&E) staining according to standard protocol. In addition, the posterior capsules were fixed with 4% paraformaldehyde for 20 min at room temperature, and then permeabilized in 0.3% Triton X-100 for 15 min at 4 °C and blocked with goat serum for 2 h at room temperature. Then, the posterior capsules were incubated with anti-mouse α-SMA antibody (1:200, Abcam, Cambridge, UK) overnight at 4 °C, and then washed three times with PBS, incubated with secondary antibody for 2 h at room temperature. The nuclei of the cells were stained with DAPI for 15 min. The images were captured by a fluorescence microscope.

### 2.11 Statistical analysis

All the statistical analyses were performed with SPSS software (version 23.0, IBM SPSS Inc., Chicago, IL, US). Statistical differences were determined using double-sided Student's t-test, Mann-Whitney *U* test, and one-way ANOVA. A p-value less than 0.05 indicated a statistically significant difference.

## 3 Results

### 3.1 TGF-β2 promotes cell migration and EMT in LECs

**Fig. 1:**
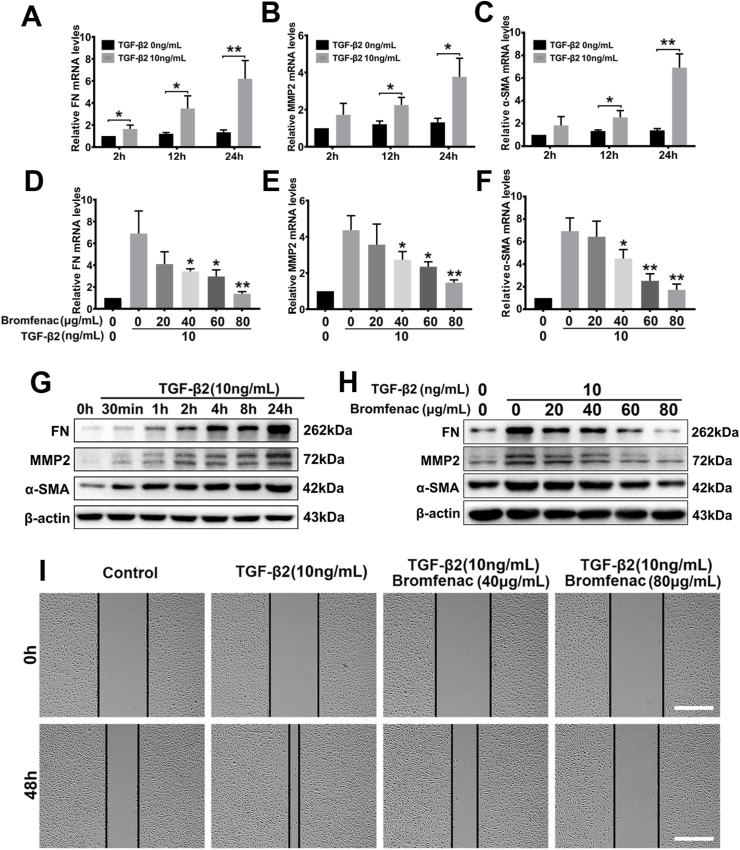
Effects of bromfenac on TGF-β2-induced EMT and cell migration in HLEC-B3 cells: the FN (A), MMP2 (B), and α-SMA (C) mRNA expression induced by 10 ng/mL TGF-β2 at 2 h, 12 h, and 24 h, measured by qPCR (*p < 0.05, **p < 0.01 vs TGF-β2 free group); effects of bromfenac in different concentrations on the TGF-β2-induced upregulation of FN (D), MMP2 (E), and α-SMA (F) mRNA expression measured by qPCR (*p < 0.05, **p < 0.01 vs TGF-β2 treated alone group); (G) FN, MMP2, and α-SMA protein expression induced by 10 ng/mL TGF-β2 for different time points detected by Western blot; (H) effects of bromfenac in different concentrations on the TGF-β2-induced upregulation of FN, MMP2, and α-SMA protein expression detected by Western blot. The cells were pre-treated, with or without bromfenac, for 24 h before induced by 10 ng/mL TGF-β2 for 24 h. (I) the wound-healing assay showed the effects of bromfenac in different concentrations on TGF-β2-induced cell migration (scale bar, 500 μm; n = 3; error bars represent SEM).

To observe the EMT markers' expression accurately, RNA expression changes need to be detected. Therefore, RNA extraction and qPCR are necessary. In this study, in order to study the effect of TGF-β2 on the EMT in HLEC-B3 cells and explore the time points at which the strongest effects on different downstream products, the cells were stimulated by 10 ng/mL TGF-β2 and the mRNA and protein levels of FN, MMP2, and α-SMA were detected by real-time qPCR and western blotting, respectively. The results showed that the mRNA levels of FN (4.70 ± 0.64 folds, p = 0.007, 24 h), MMP2 (2.87 ± 0.26 folds, p = 0.014, 24 h), and α-SMA (5.03 ± 0.54 folds, p = 0.001, 24 h) in HLEC-B3 cells upregulated significantly by TGF-β2 compared with the control group, and the best time points of stimulation of TGF-β2 for mRNA expression levels were at 24 h (Fig. 1A, B, and 1C). Correspondingly, the protein expression levels of FN (2.32 ± 0.14 folds, p = 0.017, 24 h), MMP2 (3.12 ± 0.33 folds, p = 0.035, 24 h), and α-SMA (3.21 ± 0.10 folds, p = 0.003, 24 h) increased significantly after stimulation by TGF-β2 at 24 h (Fig. 1G and Figs. S1A, S1B, S1C).

To verify the effect of TGF-β2 on the morphological changes and migration of HLEC-B3 cells, we divided the cells into two groups, treated with or without 10 ng/mL TGF-β2. The morphological changes in the cells and the scratch wound areas were observed using an inverted microscope for the two time points of 0 h and 48 h. As shown in Fig. 1I, the wound area of the TGF-β2 group was much smaller than that of the control group, which indicated that TGF-β2 effectively increased the migration ability of HLEC-B3 cells. Interestingly, no drastic morphological change was observed in this test.

### 3.2 Effects of bromfenac on TGF-β2-induced cell migration and EMT

**Fig. 2:**
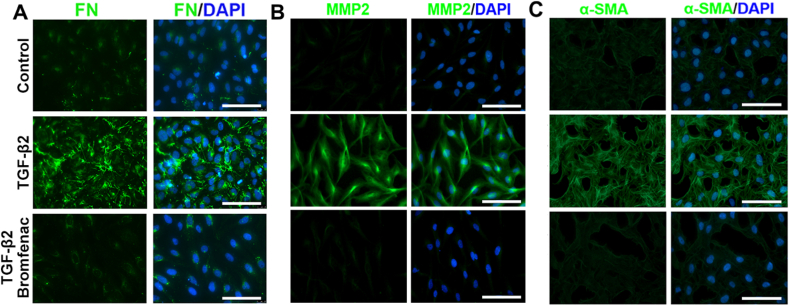
Immunofluorescence staining of the change of cellular EMT markers: 80 μg/mL bromfenac inhibited the upregulation of FN (A), MMP2 (B), and α-SMA (C) induced by 10 ng/mL TGF-β2 in HLEC-B3 cells (scale bar, 100 μm).

Bromfenac, an NSAID, has been used for the treatment of ocular inflammation since 2009. To investigate its effect on EMT and migration in HLEC-B3 cells, the cells were treated with different concentrations of bromfenac for 24 h. The cells were then stimulated by 10 ng/mL TGF-β2 for 24 h. The effect of TGF-β2 on the morphological changes and migration behaviors of HLEC-B3 cells was investigated by cell migration tests. The morphological changes in the cells and the scratch wound areas were observed using an inverted microscope for the two time points of 0 h and 48 h. The wound-healing assay showed that the remaining scratch wound areas in the bromfenac group were narrower than those in the non-bromfenac groups, indicating that bromfenac significantly suppressed TGF-β2-induced cell migration in HLEC-B3 with no morphological changes were detected (Fig. 1I). The TGF-β2-induced upregulation of FN, MMP2, and α-SMA mRNA and protein expression levels were also effectively inhibited by bromfenac. At a concentration of 40 μg/mL, bromfenac exhibited inhibition of the over-expression of EMT markers. The mRNA and protein expression of FN decreased to 0.53 ± 0.07 folds (p = 0.044) and 0.65 ± 0.27 folds (p = 0.066), respectively, compared with the group treated with TGF-β2 alone (Fig. 1D and H, and Fig. S1D). The mRNA and protein expression of MMP2 decreased to 0.63 ± 0.05 folds (p = 0.037) and 0.71 ± 0.06 folds (p = 0.013), respectively (Fig. 1E and H, and Fig. S1E). Similarly, the mRNA and protein expression of α-SMA decreased to 0.65 ± 0.01 folds (p = 0.041) and 0.84 ± 0.03 folds (p = 0.048), respectively (Fig. 1F and H, and Fig. S1F). Further, the mRNA and protein expression of FN decreased to 0.21 ± 0.03 folds (p = 0.010) and 0.39 ± 0.09 folds (p = 0.011) when treated with 80 μg/mL bromfenac (Fig. 1D and H, and Fig. S1D). The mRNA and protein expression of MMP2 decreased to 0.35 ± 0.04 folds (p = 0.004) and 0.40 ± 0.11 folds (p = 0.006), respectively (Fig. 1E and H, and Fig. S1E). Also, the mRNA and protein expression of α-SMA decreased to 0.25 ± 0.02 folds (p = 0.002) and 0.64 ± 0.05 folds (p = 0.004), respectively (Fig. 1F and H, and Fig. S1F). Since bromfenac at a concentration of 80 μg/mL, compared to 40 μg/mL, was more able to suppress the expression of EMT markers to a level similar to that of no treatment group, only a concentration of 80 μg/mL bromfenac was chose for immunofluorescence assay. As shown in the immunofluorescent images (Fig. 2), the fluorescence intensity of FN, MMP2, and α-SMA decreased significantly treated with the bromfenac compared with the groups treated with TGF-β2 alone. In addition, Fig. 2C showed that the cells underwent morphological changes when treated with TGF-β2 alone. However, the cells treated with 80 μg/mL bromfenac and TGF-β2 maintained an epithelial phenotype. Collectively, these results indicated that bromfenac could inhibit TGF-β2-induced EMT in HLEC-B3 and showed a concentration-dependent inhibition.

### 3.3 Effects of bromfenac on the TGF-β2-activated ERK/GSK-3β signaling pathway

**Fig. 3:**
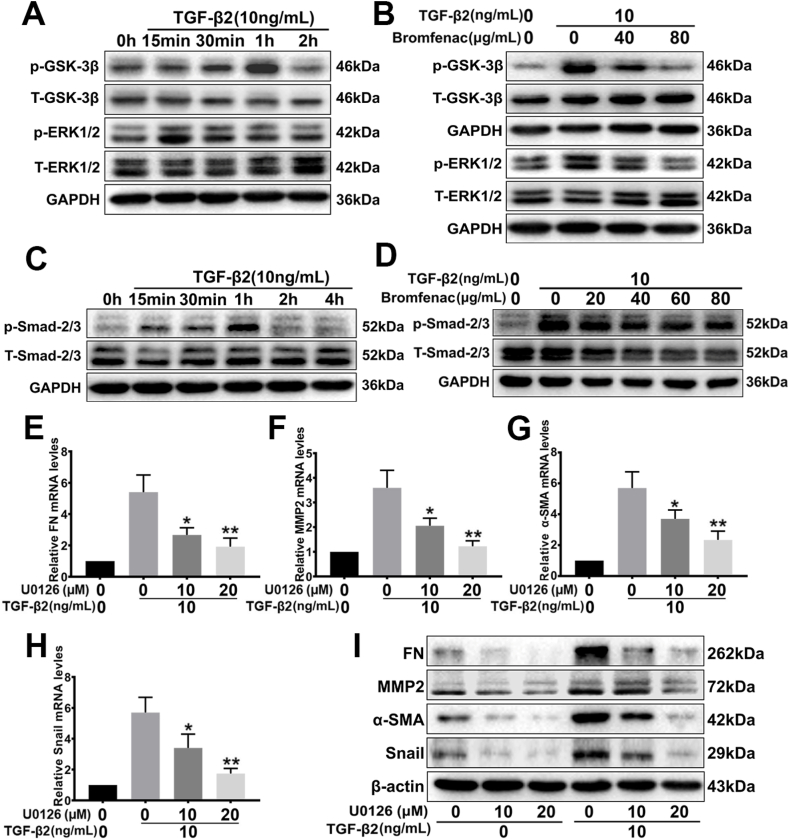
Effects of bromfenac on the TGF-β2-activated ERK/GSK-3β pathway in HLEC-B3. U0126 suppressed TGF-β2-induced up-regulation of the EMT markers FN, MMP2, α-SMA, and Snail. (A) TGF-β2 stimulated the phosphorylation of ERK1/2 and GSK-3β, and (B) bromfenac significantly inhibited phosphorylation. The cells were pre-treated with or without bromfenac for 24 h before induced by 10 ng/mL TGF-β2. (C) TGF-β2 stimulated the phosphorylation of Smad2/3, but (D) bromfenac could not inhibit phosphorylation; and pre-treatment with U0126 for 2 h suppressed the TGF-β2-induced upregulation of FN (E), MMP2 (F), α-SMA (G), and Snail (H) mRNA, and protein expression (I) (*p < 0.05, **p < 0.01 vs TGF-β2 treated alone group, n = 3, error bars represent SEM).

The processes of TGF-β2-induced lens epithelial cell migration and EMT are associated with multiple signaling pathways, which include canonical TGF-β/Smad signaling and TGF-β/ERK/GSK-3β signaling. To validate the activation of TGF-β2 *via* these signaling pathways, we tested the phosphorylation levels of Smad2/3, EKR1/2, and GSK-3β at different time points in HLEC-B3 cells after stimulation by 10 ng/mL of TGF-β2. Fig. 3A and C showed that TGF-β2 activated ERK1/2 by phosphorylating rapidly in 15 min (1.75 ± 0.07 folds, p = 0.012, Fig. S2A), and activated Smad2/3 at 1 h (6.96 ± 0.62 folds, p = 0.016, Fig. S3A). TGF-β2 was also able to stimulate the phosphorylation of GSK-3β at 1 h (3.71 ± 0.25 folds, p = 0.012, Fig. 3A and Fig. S2B). Notably, phosphorylation of GSK-3β at Serine 9 would lead to the inactivation of GSK‐3β [37]. These results suggested that TGF-β2 could activate ERK1/2 and Smad2/3 signaling.

To explore the mechanism by which bromfenac inhibited the EMT of HLEC-B3 cells induced by TGF-β2, the total protein was extracted at 15 min and 1 h TGF-β2 stimulation time points after pre-treatment for 24 h with different concentrations of bromfenac, respectively. The phosphorylation levels of Smad2/3, EKR1/2, and GSK-3β were detected by Western blot analysis. We found that bromfenac could significantly inhibit the TGF-β2-induced phosphorylation of ERK1/2 and GSK-3β at concentrations of ≥40 μg/mL. Treated with 40 μg/mL bromfenac, the relative expression of p-EKR1/2 and p-GSK-3β decreased to 0.58 ± 0.07 folds (p = 0.042) and 0.65 ± 0.11 folds (p = 0.040), respectively, compared with the group treated with TGF-β2 alone. When the concentration of bromfenac reached 80 μg/mL, the inhibition of p-EKR1/2 (0.46 ± 0.03 folds, p = 0.031) and p-GSK-3β (0.53 ± 0.14 folds, p = 0.032) relative expression was more significant (Fig. 3B and Figs. S3C and S3D). However, bromfenac did not inhibit the phosphorylation of Smad2/3 (Fig. 3D and Fig. S3B). Collectively, these results suggested that bromfenac inhibits the expression of EMT markers induced by TGF-β2 in HLEC-B3 cells might *via* the ERK/GSK-3β signaling pathway, but not *via* the canonical TGF-β/Smad pathway.

### 3.4 The role of the ERK/GSK-3β pathway in the TGF-β2-induced EMT of LECs

The above results proved that TGF- β2 could not only induce upregulation of EMT markers and the related transcription factor Snail but also stimulate the phosphorylation of ERK1/2 in LECs. To verify the relationship between ERK signaling and the target genes FN, MMP2, α-SMA, and Snail, each group pre-treated with MEK-specific inhibitor U0126 (10 μM or 20 μM) were stimulated with 10 ng/mL TGF-β2, then the mRNA and protein expressions of Snail after 2 h, and FN, MMP2, and α-SMA after 24 h were detected.

**Fig. 4:**
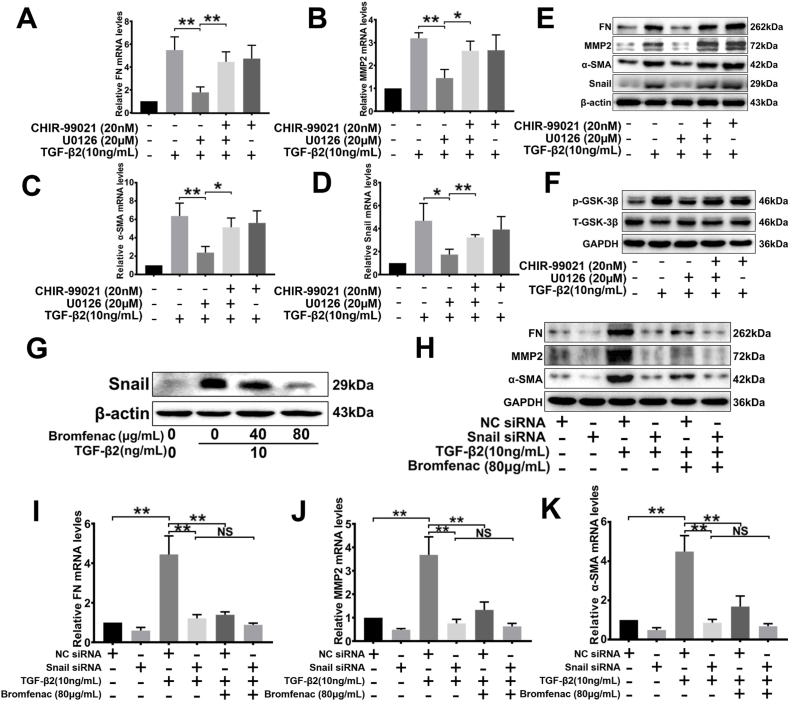
Effects of U0126 and CHIR-99021 on TGF-β2-induced EMT in HLEC-B3 and the role of Snail in TGF-β2-induced EMT. The cells were treated with 20 nM CHIR-99021 for 24 h and 20 μM U0126 for 2 h before 10 ng/mL TGF-β2 treatment. The mRNA (A, B, C, and D) and protein (E) expression of FN, MMP2, α-SMA, and Snail detected by qPCR and Western blot analysis, respectively (*p < 0.05, **p < 0.01 between groups); (F) the TGF-β2-induced phosphorylation of GSK-3β after treatment with CHIR-99021 and U0126 and evaluated by Western blot analysis. The cells were pre-treated with or without bromfenac for 24 h before induced by 10 ng/mL TGF-β2, and Snail expression (G) was detected by Western blot analysis. The cells were transfected with Snail siRNA or NC siRNA before bromfenac and 10 ng/mL TGF-β2 treatment. The protein (H) and mRNA (I, J, and K) expression of FN, MMP2, and α-SMA detected by qPCR and Western blot analysis, respectively (*p < 0.05, **p < 0.01 between groups). NS represents no significance compared with the Snail siRNA + TGF-β2 treated alone group (n = 3, error bars represent SEM).

It was found that MEK-specific inhibitor U0126 could significantly suppress the mRNA and protein expressions of EMT markers FN, MMP2, and α-SMA in a concentration-dependent manner, particularly when the expressions were stimulated with TGF-β2 (Fig. 3E, F, 3G and 3I). Treated with 20 μM U0126, the protein expression of FN, MMP2, and α-SMA decreased to 0.27 ± 0.02 folds (p = 0.004), 0.32 ± 0.02 folds (p = 0.004), and 0.19 ± 0.01 folds (p = 0.008) respectively, compared with the group treated with TGF-β2 alone (Fig. 3I and Figs. S4A, S4B, S4C). Similarly, Snail mRNA (0.31 ± 0.01 folds, p = 0.003) and protein (0.19 ± 0.02 folds, p = 0.022) expression could also be suppressed by 20 μM U0126 (Fig. 3H and I, and Fig. S4D). Afterward, the cells were treated with dual inhibitors, U0126 (20 μM) and CHIR-99021 (GSK-3 inhibitor, 20 nM), to investigate the relationship between GSK-3β and ERK signaling. U0126 effectively inhibited the phosphorylation of GSK-3β induced by TGF-β2; however, CHIR-99021 antagonized the inhibition of U0126 (Fig. 4F and Fig. S5E). Treated with U0126 and CHIR-99021 simultaneously, the expression of EMT markers increased significantly compared with the group treated with U0126 alone (Fig. 4A, B, 4C, 4D, and 4E). It was found that the GSK-3 inhibitor upregulated the expression of FN (1.45 ± 0.06 folds, p = 0.037, Fig. 4E and S5A), MMP2 (1.54 ± 0.16 folds, p = 0.026, Fig. 4E and S5B), α-SMA (1.82 ± 0.20 folds, p = 0.006, Fig. 4E and S5C), and Snail (2.07 ± 0.27 folds, p = 0.015, Fig. 4E and S5D). In addition, CHIR-99021 kept GSK-3β at a high phosphorylation level when used alone with TGF-β2, which inhibited the activity of GSK-3β substantially (Fig. 4F and Fig. S5E). Therefore, blocking ERK signaling with U0126 could repress the TGF-β2-induced EMT of LECs, but the inhibition of GSK-3β activity could significantly diminish the effect of U0126. Taken together, these results indicated that the TGF-β2-induced over-expression of EMT markers in LECs was regulated *via* ERK1/2 signaling, and GSK-3β played an indispensable regulatory role in the signaling pathway (ERK/GSK-3β signaling pathway).

### 3.5 Effect of siRNA knockdown of Snail on TGF-β2-induced EMT in LECs

Transfection is a non-viral technology for delivering foreign nucleic acids into eukaryotic cells and studying the function of specific genes. By knocking down specific genes with siRNA transfection, the role of genes can be defined accurately. In this study, siRNA transfection was used to knock down the Snail and explore how the ERK/GSK-3β signaling pathway regulated the TGF-β2-induced over-expression of EMT markers. 1.5 × 10^5^ HLECs were seeded in 6-well plates and transfected with Snail siRNA or NC siRNA after 24 h, then induced with TGF-β2 and analyzed using qPCR and Western blot analysis. Knockdown of Snail effectively inhibited the TGF-β2-induced over-expression of FN, MMP2, and α-SMA mRNA, and protein levels (Fig. 4H, I, 4J, 4K, and Figs. S6B, S6C, S6D).

As mentioned above, the TGF-β2-induced upregulation of FN, MMP2, and α-SMA could be effectively inhibited by bromfenac at a concentration of 80 μg/mL. Thus, two groups of cells were pre-treated with bromfenac before being treated with TGF-β2. In the groups with NC siRNA transfection, bromfenac significantly inhibited the TGF-β2-induced over-expression of FN (0.53 ± 0.10 folds, p = 0.028, Fig. 4H and S6B), MMP2 (0.51 ± 0.07 folds, p = 0.044, Fig. 4H and S6C), and α-SMA (0.36 ± 0.04 folds, p = 0.003, Fig. 4H and S6D), while in the groups with Snail siRNA transfection, there was no significant difference in the expression of FN (0.84 ± 0.08 folds, p = 0.223, Fig. 4H and S6B), MMP2 (0.66 ± 0.07 folds, p = 0.105, Fig. 4H and S6C), and α-SMA (1.22 ± 0.08 folds, p = 0.147, Fig. 4H and S6D) between the Snail siRNA + TGF-β2 group and the Snail siRNA + TGF-β2+ bromfenac group. In addition, the expression of Snail mRNA and protein was detected after the cells were treated with bromfenac and TGF-β2. The results showed that the 40 μg/mL and 80 μg/mL bromfenac could decrease the expression of Snail to 0.65 ± 0.05 folds (p = 0.026) and 0.47 ± 0.04 folds (p = 0.009), respectively, compared with the group treated with TGF-β2 alone (Fig. 4G and Fig. S6A). Taken together, these results suggested that the transcription factor Snail is an indispensable downstream mediator involved in the inhibition of TGF-β2-induced EMT by bromfenac, and they also verified that the inhibition effect of bromfenac on TGF -β2-induced EMT in LECs was achieved by blocking the ERK/GSK-3β/Snail signaling pathway.

### 3.6 Bromfenac inhibits TGF-β2-induced EMT in human anterior capsules

**Fig. 5:**
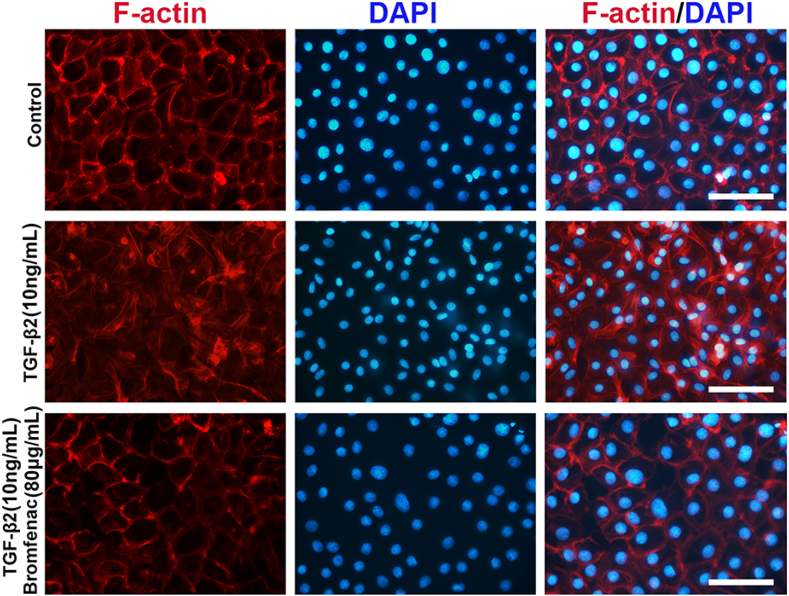
Effects of bromfenac on TGF-β2-treated human anterior capsules. The capsules were incubated with TGF-β2 and bromfenac, and assessed using F-actin staining with fluorescent Phalloidin (scale bar, 100 μm).

A primary lens epithelial cell model using human anterior capsules was established to further verify the inhibitory effect of bromfenac on TGF-β2-induced EMT [38]. This model was advantageous for PCO research since it maintained many of the important markers and functions of cells as seen *in vivo* and provided a culture environment for cells with natural matrices. After a total of 48 h of culturing *ex vivo*, we observed that the cells, treated with 10 ng/mL TGF-β2 alone, lost their regular epithelial phenotype appearance, and the F-actin in this group was rearranged into stress filaments. However, the cells, treated with 10 ng/mL TGF-β2 and 80 μg/mL bromfenac, still maintained the epithelial phenotype (Fig. 5). This result indicated that the F-actin arrangement was closely regulated by TGF-β2 during EMT processes, and bromfenac could also suppress TGF-β2-induced EMT in primary LECs*.*

### 3.7 Surface characterization and drug-release kinetics of bromfenac-eluting IOLs

**Fig. 6:**
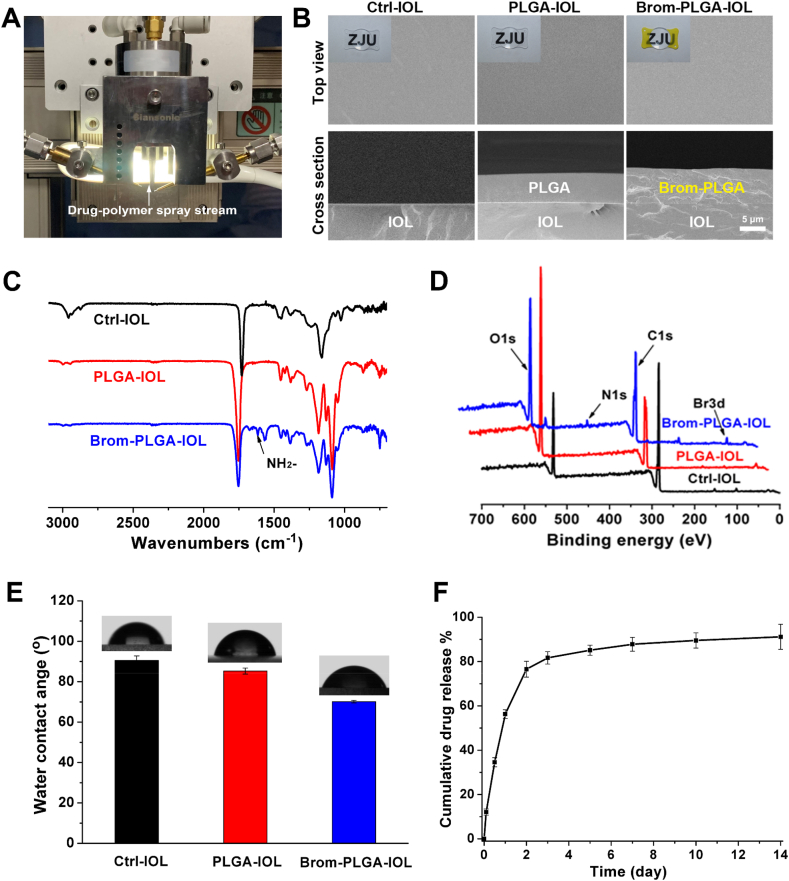
Surface morphology and drug-release kinetics of drug-eluting IOLs: (A) schematic diagram of drug-eluting IOL construction; (B) bright field and SEM photographs of drug-eluting IOLs. SEM images showed that the thickness of the films on the bromfenac-PLGA-IOLs was ~6 μm; slightly thicker than that on the PLGA-IOLs, at ~5 μm (scale bar, 5 μm); ATR-FTIR spectra (C), XPS patterns (D), and water contact angle (E) of Ctrl-IOL, PLGA-IOL, and Brom-PLGA-IOL; (F) drug-release kinetics of bromfenac-eluting IOLs. Nearly 81.7% of the bromfenac was released in 72 h and 91.2% of bromfenac was released over 14 days.

To better investigate the therapeutic effect and the mechanism of bromfenac in PCO inhibition, bromfenac-eluting IOLs with sustained drug release properties were prepared using advanced ultrasonic spraying technology owing to the ability of precise spray position control [39,40]. The coating was only sprayed onto the plate haptics of IOLs with an efficiency of approximately 100% to ensure the transparency and clarity of IOLs to the greatest extent. SEM images showed the thickness of the film on the bromfenac-PLGA-IOL was ~6 μm, slightly thicker than that on the PLGA-IOL (~5 μm; Fig. 6B). In addition, all the surfaces of the films were smooth and had almost no pores, which was beneficial for controlling the burst release of drugs. The chemical structure of the coating's surface was characterized by ATR-FTIR and XPS (Fig. 6C and D). ATR-FITR and XPS results showed that the Brom-PLGA-IOL appeared the characteristic peak of the NH_2_- group at 1630 cm^−1^ and the signal of Br3d at 69.0 eV from bromfenac, respectively. The surface wettability of the coating on IOLs was measured by water contact angle (Fig. 6E). The Ctrl-IOL that made of hydrophobic acrylic had a water contact angle of 90.6 ± 2.0°. After being coated with PLGA or PLGA with bromfenac, the water contact angle of the coating decreased to 85.3 ± 1.5° or 70.1 ± 0.6°, respectively, with improved surface hydrophilicity. All these results verified the successful load of the drug on IOLs. Meanwhile, UV–vis spectra indicated that Brom-PLGA-IOL kept the same transmittance as Ctrl-IOL in the optical center since the drug was only sprayed onto the plate haptics of IOLs, which could ensure the smoothness and transparency of the IOLs' optics (Fig. S7). The bromfenac release behaviors of the films were then investigated in an aqueous humor-mimicking solution. Nearly 81.7% of the bromfenac was released in 72 h, and 91.2% of the bromfenac was released over 14 days (Fig. 6F). More importantly, all the bromfenac in the coatings was released after PLGA degraded completely in 2 months; therefore, it is likely that the film of a bromfenac-eluting IOL can perform sustained bromfenac release in aqueous humor.

### 3.8 Bromfenac-eluting IOLs for PCO prevention *in vivo*

**Fig. 7:**
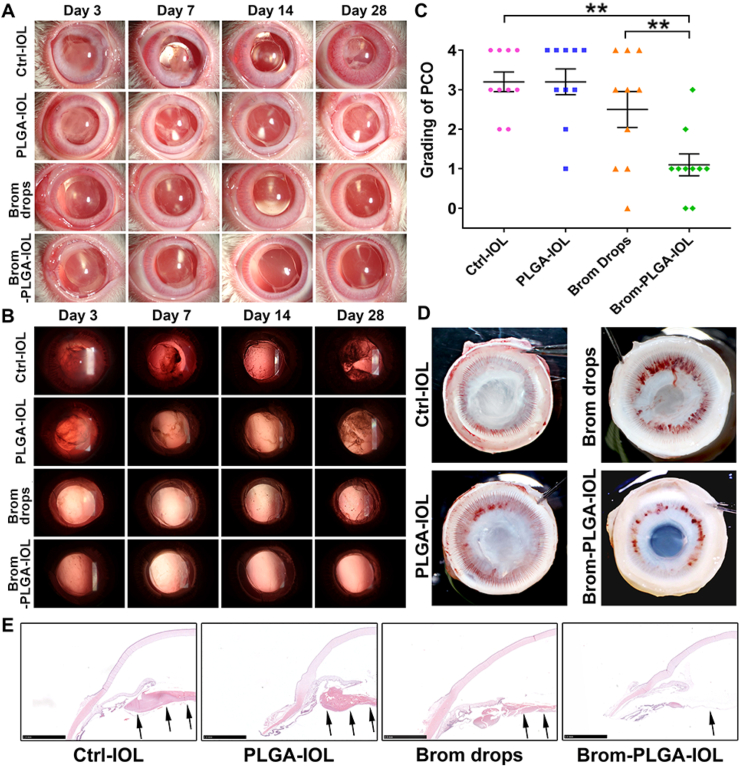
Postoperative assessment of PCO in the rabbit models: anterior segment structures (A) and posterior capsules (B), evaluated using a slit lamp photography system on the third, seventh, fourteenth, and twenty-eighth days after surgery; (C) Grading of PCO development of the eyes of the four groups (**p < 0.01 vs control-IOL or Brom drops group, n = 10, error bars represent SEM); (D) Miyake-Apple view of the eyes of the four groups. (E) Histological examination of the anterior ocular segment of the rabbit eyes from each group. The black arrows show that posterior capsules, with few cells, remained smooth in the bromfenac-PLGA-IOL group, while a large amount of proliferative material accumulated on the posterior capsules in the other three groups (scale bar, 2.5 mm).

The PCO prevention effects of bromfenac-eluting IOLs were subsequently investigated in rabbit PCO models. The anterior segments of rabbit eyes were evaluated using a slit lamp photography system on the third, seventh, fourteenth, and twenty-eighth days after surgery. In the control-IOL (Ctrl-IOL) and PLGA-IOL groups, progressively more proliferative material accumulated on the posterior capsules from the fourteenth day after surgery onwards, while the posterior capsules of the bromfenac-PLGA-IOL group remained smooth (Fig. 7A and B). In the control-IOL with bromfenac eye drops (Brom drops) group, the proliferative material in the posterior capsule was less than those in the control-IOL and PLGA-IOL groups. However, it was still significantly more than that in the bromfenac-PLGA-IOL group. Fig. 7A also showed that the postoperative short-term anterior chamber inflammation in the bromfenac-PLGA-IOL group and Brom drops group were milder than those in the other two groups. Fig. 7C and Table 2 showed the grading of PCO development. In the bromfenac-PLGA-IOL group, eight of ten rabbits had a PCO rated as grade 0 or 1, whereas in the other three groups, at least six of ten rabbits in each group had PCO rated as grade 3 or above. The average grade of the bromfenac-PLGA-IOL group was 1.10 ± 0.26, better than that of the control-IOL group (3.20 ± 0.24), PLGA-IOL group (3.20 ± 0.31), and Brom drops group (2.10 ± 0.41). There was a significant difference between the groups (NPar test: Kruskal-Wallis test: χ2 = 15.421, p = 0.001). At the end of the experiment, the Miyake-Apple view of the eyes showed no prominent visible proliferative tissues on the posterior capsules of the bromfenac-PLGA-IOL group (Fig. 7D). Still, many proliferative fibrin tissues were deposited on the posterior capsules of the other three groups. Notably, compared with the control-IOL group, the PCO development was also suppressed to some extent in the Brom drops group, but without a statistical difference (Mann-Whitney *U* test, p = 0.353). This illustrated that administration by the eye drop route could effectively control postoperative anterior chamber inflammation but had limited efficacy for PCO prevention due to its low bioavailability to intraocular tissues. The histological examination and immunofluorescence assay of the posterior capsules showed different manifestations for the bromfenac-PLGA-IOL and other groups. There were few cells on the posterior capsules in the bromfenac-PLGA-IOL group. However, a large amount of proliferative material accumulated on the capsules and the Soemmering's ring formations in the other three groups (Fig. 7E). Moreover, the bromfenac-PLGA-IOL group displayed a much weaker fluorescence intensity of α-SMA, an EMT marker, than the other three groups on the posterior capsules, indicating the high efficiency of bromfenac in the drug-eluting IOL (Fig. S8).

**Table 2:** Grading of PCO development.

Grades	Ctrl-IOL	PLGA-IOL	Brom drops	Brom-PLGA-IOL
0 = None	0	0	1	2
1 = Slight	0	1	2	6
2 = Moderate	2	1	1	1
3 = Pronounced	4	3	3	1
4 = Severe	4	5	3	0

Data represent the number of cases.

**Fig. 8:**
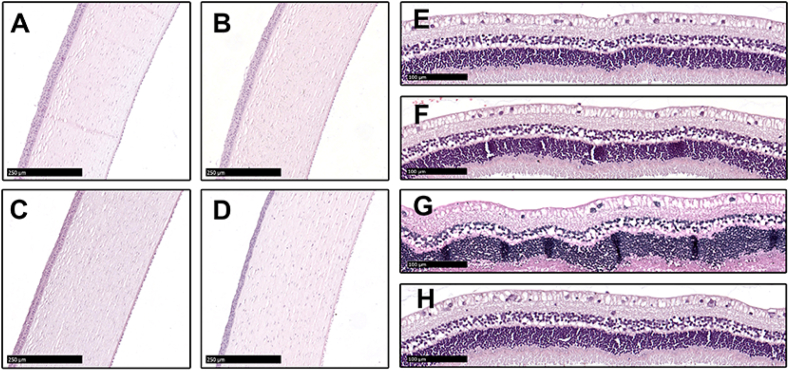
Histological analysis of H&E staining of corneas and retinas. A and E: control-IOL group; B and F: PLGA-IOL group; C and G: Brom drops group; D and H: bromfenac-PLGA-IOL group. A, B, C, and D show that the corneas from all groups are smooth and intact (scale bar, 250 μm). E, F, G, and H show that the retinas in the eyes of all the groups did not significantly differ in their morphology (scale bar, 100 μm).

The corneas, irises, and retinas in the eyes of the bromfenac-PLGA-IOL group were not significantly different from those of the other three groups in their morphology. All the tissues were rendered intact and neat structures with no significant damage. The cornea presented five continuous well-defined layered structures without inflammatory cells infiltration, and no edema and other structural abnormalities were observed in the retina of all groups. Following cataract extraction with posterior chamber IOLs implantation, there was no death or abnormal behavior in any experimental animal groups during the study period during all periods of the *in vivo* study (Figs. 7E and 8). The potential topical and systemic untoward effects were examined by experienced technicians skilled in discriminating between normal and abnormal animal behavior. Overall, no apparent toxicity was observed and no morphological or behavioral changes were found after twenty-eighth-day exposure, confirming the high biocompatibility and safety of the drug-eluting IOLs, which provided the foundation for future clinical application.

## 4 Discussion

The present study revealed the potential mechanism of the NSAID, bromfenac, in inhibiting the TGF-β2-induced EMT in LECs, and also reported a novel and feasible approach for PCO prevention based on bromfenac-eluting IOLs. It is the first report that NSAIDs can inhibit TGF-β2-induced LECs migration and EMT *via* ERK/GSK-3β signaling pathway with the transcription factor Snail as the indispensable role in this process. Although some IOLs with drug coating by ultrasonic spray technology were reported [41,42], these novel IOLs were only proved *in vitro* and never confirmed *in vivo*. Brom-PLGA-IOL displayed excellent PCO inhibition effects in the rabbit PCO models.

PCO mainly comes from the proliferation, migration, fibrosis, and EMT of LECs in the lens capsule after stimulation by several growth factors and cytokines [43]. The development of anterior subcapsular cataract (ASC) and PCO closely relates to the EMT of LECs triggered by a diverse set of stimuli, of which the TGF-β2 growth factor is one of the most [44,45]. Previous studies demonstrated that TGF-β2 played an essential role in the formation of PCO *via* the canonical TGF-β/Smad signaling pathway, as well as *via* non-canonical signaling [20,46]. Activation of the ERK signaling pathway was involved in cell carcinogenesis, cancer cell metastasis, and a series of fibrotic diseases, including PCO [26,47,48]. Our study revealed the critical role of the ERK signaling pathway in the TGF-β2-induced EMT of human LECs. We also found that TGF-β2 treatment inactivated GSK-3β through phosphorylation *via* ERK signaling. Moreover, GSK-3β regulated the expression of the downstream transcription factor Snail, contributing to the production of cell migration and EMT-related protein. Therefore, the study concluded that ERK/GSK-3β/Snail signaling played a crucial role in the regulation of TGF-β2-induced cell migration and EMT in LECs.

Snail is a highly conserved transcription factor and a key moderator of EMT-specific protein and ECM synthesis in tumor progression and metastasis [[49], [50], [51]]. It also played a crucial role in the advancement of TGF-β2-induced EMT in LECs, whose transcription factor was moderated by GSK-3β. Previous studies demonstrated that GSK-3β was a protein kinase closely related to cell proliferation, apoptosis, migration, and transdifferentiation, as well as the regulation of glucose [21,22,52]. The present study found that TGF-β2 activated the ERK pathway and the phosphorylation of GSK-3β, and blocking the ERK pathway with U0126 could suppress the phosphorylation of GSK-3β significantly. Therefore, the ERK pathway was necessary for the TGF-β2-induced inactivation of GSK-3β. In addition, this study verified the regulatory effect of the ERK/GSK-3β signaling pathway on the expression of transcription factor Snail by treating cells with GSK-3β inhibitor; furthermore, the siRNA-mediated silencing of the Snail gene directly down-regulated the expression of EMT-specific proteins, such as FN, MMP2, and α-SMA.

Bromfenac is a topical ophthalmic non-steroidal anti-inflammatory drug that can effectively inhibit the production of prostaglandins, mainly by blocking COX-2, and thus reduce inflammation and prevent pseudophakic cystoid macular edema after cataract surgery [53,54]. Bromfenac inhibits COX-2 *in vitro* roughly four to five times more effective than other ophthalmic NSAIDs [55]. This study showed that bromfenac significantly inhibited the TGF-β2-induced high expression of Snail, FN, MMP2, and α-SMA, and this inhibition could be substantially limited by Snail siRNA transfection. Interestingly, we found that bromfenac could efficiently suppress the phosphorylation of ERK1/2 and GSK-3β induced by TGF-β2, while the phosphorylation of Smad2/3 was not affected. Smad is a transduction intermediate and activin receptor, which can regulate the transcription of Snail by binding to its promoter directly [56,57]. TGF-β-activated Smad could be phosphorylated and recruited by MAPK/ERK-activated RAS responsive element-binding protein 1 (RREB1) to Snail [58,59]. In this study, however, the phosphorylation of Smad2/3 was not inhibited by bromfenac, and therefore, a conclusion could be drawn that Snail is a critical component in the process of bromfenac inhibiting the TGF-β2-induced upregulation of FN, MMP2, and α-SMA expression, and the down-regulation of Snail results from a direct effect on the Smad-independent ERK/GSK-3β/Snail signaling pathway.

EMT is characterized by a loss of epithelial properties and acquisition of a mesenchymal phenotype, associated with increased cellular motility and rearrangement of the actin cytoskeleton [60]. TGF-β2 stimulated the activation of the ERK pathway, thus facilitating the migration ability of LECs; however, bromfenac significantly suppressed TGF-β2-induced migration in LECs. Moreover, immunofluorescent images stained with phalloidin showed that bromfenac could also effectively inhibit the TGF-β2-induced reorganization and polymerization of F-actin in the primary LECs of human anterior capsules. It is worth noting that the Soemmering's ring formations, a feature of fibrosis rather than EMT, usually appeared in the late stage of PCO development. PCO occurred in this study as early as 14 days after surgery with Soemmering's ring formation. Hence, a duration of 28 days is adopted to assess PCO in rabbits [6,61]. Soemmering's ring in the bromfenac-PLGA-IOL group were much less than that in other groups(Fig. 7B), which may come from the anti-fibrotic properties of bromfenac in PCO prevention [62,63].

PLGA has been approved by FDA for its use in drug delivery systems, including ophthalmic implantation (Ozurdex®), due to its excellent biocompatibility and adjustable biodegradability [32,33,[64], [65], [66], [67]]. In this study, each IOL firmly loaded about 100 μg of bromfenac in PLGA coatings. The coatings of the IOL would not crack or shed, even if the IOL was heavily folded during surgery. Most importantly, the prepared biocompatible bromfenac-eluting IOL released the drug smoothly with effective PCO prevention and significant postoperative inflammation inhibition in the rabbit PCO models. The topical administration of 0.1% bromfenac eye drops four times per day after surgery could reduce the postoperative inflammation to a certain extent. Yet it showed no remarkable effect in PCO prevention in the current study because of its extremely low drug delivery efficiency, that is, the 0.1% bromfenac eye drop route failed to achieve the optimal efficacy as the estimated actual intraocular administration by the drug loading of the novel IOL. The drug delivery efficiency was less than 3% into the anterior chamber by the topical route, while approximately 100% into the posterior capsule directly through the intracameral route [29,68]. But the drug released from bromfenac-PLGA-IOL is closer to the posterior capsule than that from the eye drop route and could be exposed to the target directly, which avoids the drugs being excreted from the anterior chamber through aqueous humor outflow to a considerable extent [69]. Thus, the intraocular sustained-release could be more effective for intraocular diseases with less drug dosage. Moreover, drug-eluting IOL could mitigate the risk of the corneal melt that is the most severe side effect of topical NSAIDs, since the drug released from the intraocular IOL was able to bypass the corneal barrier and directly reach the posterior capsule. In addition, the implantation of drug-eluting IOL may also eliminate the need for long-term eye drop self-administration and improve treatment adherence.

Admittedly, there still exist some limitations in this study. First, our study indicated that bromfenac could regulate ERK/GSK-3β/Snail signaling, thus inhibiting the TGF-β2-induced EMT in LECs. However, it is also possible that bromfenac can achieve inhibition of cell migration and EMT *via* other signaling pathways. Thus, the role of bromfenac and its specific targets in these pathways should be further studied. Second, the drug release duration of drug-loaded IOLs was mainly in the range of 2–14 days on rabbit PCO models [6,61,70]. Although our prepared Brom-PLGA-IOL could release bromfenac constantly for 14 days, approximately 80% of the bromfenac was released rapidly from the drug-eluting IOLs within 2 days. Therefore, different drug release behaviors, especially longer and smoother ones from IOLs are worthy of in-depth exploration, and the relationship between drug release behaviors and therapeutic effect should also be further studied. We hope more relevant in-depth pharmacokinetic studies will be adopted as a reference for a more precise dosage of drug loading, and novel coatings with the slow and zero-order release of drugs for IOLs could be developed for clinical translation.

## 5 Conclusion

In summary, the present study showed that bromfenac could inhibit TGF-β2-induced lens epithelial cell migration and EMT through regulating ERK/GSK-3β/Snail signaling. The bromfenac-eluting IOLs were further prepared and implanted into the capsule during cataract surgery, exhibiting remarkable PCO prevention and inflammation suppression effects with excellent biocompatibility *in vivo*. These novel bromfenac-based drug-eluting IOLs offer a promising alternative for clinical application for PCO prevention.

## CRediT authorship contribution statement

**Xiaobo Zhang:** generated the idea, Writing – original draft, designed and performed the experiments, Data curation, Formal analysis. **Kairan Lai:** generated the idea, Writing – original draft, designed and performed the experiments. **Su Li:** designed and performed the experiments, Data curation, Formal analysis. **Jing Wang:** performed the experiments, Data curation, Formal analysis. **Jiayong Li:** performed the experiments, Data curation, Formal analysis. **Wei Wang:** helped provide materials and performed the in vivo experiments. **Shuang Ni:** helped provide materials and performed the experiments. **Bing Lu:** helped provide materials and performed the experiments. **Andrzej Grzybowski:** Writing – review & editing, supervised the study, revised and finalized the manuscript. **Jian Ji:** generated the idea, Writing – review & editing, supervised the study. **Haijie Han:** generated the idea, Writing – review & editing, designed the experiments, Data curation, Formal analysis, revised and finalized the manuscript. **Ke Yao:** generated the idea, Writing – review & editing, supervised the study, revised and finalized the manuscript.

## Declarations of competing interest

The authors declare that they have no financial and personal relationships with other people or organizations that could have appeared to influence the work reported in this paper.
