# Supplementary material for: Drug-eluting intraocular lens with sustained bromfenac release for conquering posterior capsular opacification
Source: Bioact Mater. 2021 Jul 23;9:343–57. doi: 10.1016/j.bioactmat.2021.07.015 (PMC8586266; doi:10.1016/j.bioactmat.2021.07.015)
Supplement: Multimedia component 1 [file mmc1.docx]

Supporting Information

**Drug-eluting intraocular lens with sustained bromfenac release for conquering posterior capsular opacification**

Xiaobo Zhang^a,^ ^b^, Kairan Lai^a, b^, Su Li^a, b^, Jing Wang^e^, Jiayong Li^a, b^, Wei Wang^a^, Shuang Ni^a^, Bing Lu^a, b^, Andrzej Grzybowski^c, d^, Jian Ji^e^, Haijie Han^a, b, #^, Ke Yao^a, b^^, #^

a Eye Center, Second Affiliated Hospital, School of Medicine, Zhejiang University, Hangzhou, P. R. China

b Zhejiang Provincial Key Laboratory of Ophthalmology, Hangzhou, P. R. China

c Department of Ophthalmology, University of Warmia and Mazury, 60-554 Olsztyn, Poland

d Institute for Research in Ophthalmology, Gorczyczewskiego 2/3, 61-553 Poznan, Poland

e MOE Key Laboratory of Macromolecule Synthesis and Functionalization of Ministry of Education, Department of Polymer Science and Engineering, Zhejiang University, Hangzhou, P. R. China

^#^**Corresponding author:**

E-mail address: [xlren@zju.edu.cn](mailto:xlren@zju.edu.cn) (K. Yao); [hanhj90@zju.edu.cn](mailto:hanhj90@zju.edu.cn) (H. Han)

**Supplementary Figures**


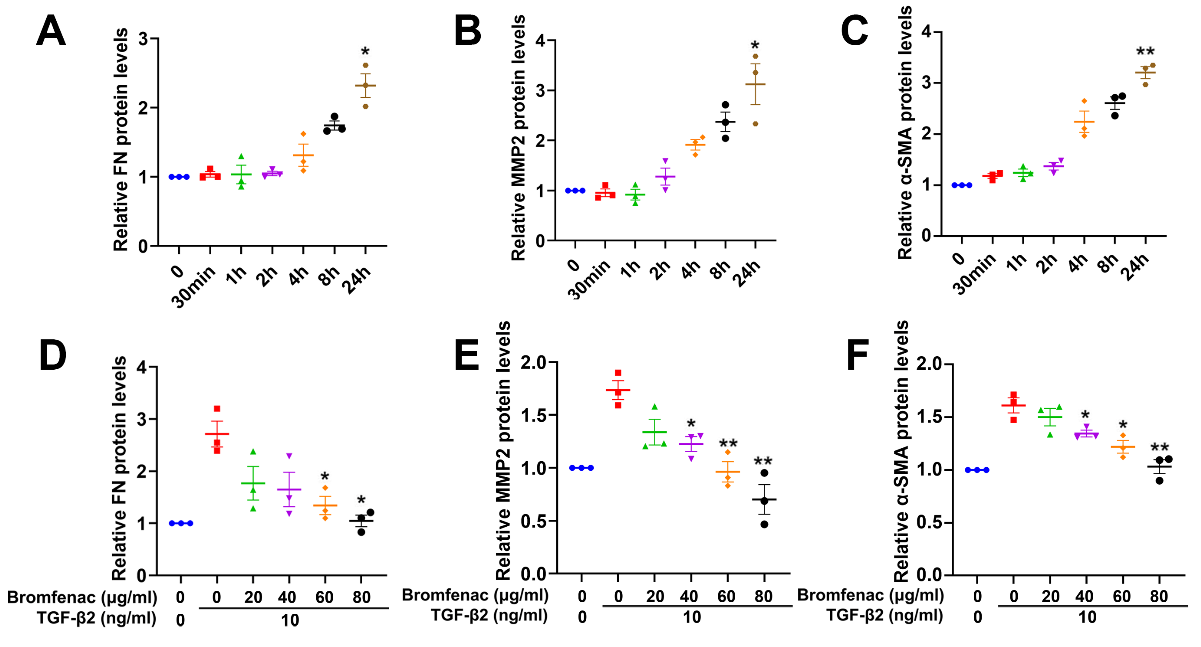


**Figure S1.** Quantification of FN, MMP2, and α-SMA protein levels by western blotting in HLEC-B3: (A) the protein expression of FN (corresponding to Fig. 1G) induced by TGF-β2 for different time point detected; (B) the protein expression of MMP2 (corresponding to Fig. 1G) induced by TGF-β2 for different time point detected; (C) the protein expression of α-SMA (corresponding to Fig. 1G) induced by TGF-β2 for different time point detected (*p < 0.05, **p < 0.01 vs TGF-β2 free group); (D) effects of bromfenac in different concentrations on the TGF-β2-induced upregulation of FN (corresponding to Fig. 1H) protein expression; (E) effects of bromfenac in different concentrations on the TGF-β2-induced upregulation of MMP2 (corresponding to Fig. 1H) protein expression; (E) effects of bromfenac in different concentrations on the TGF-β2-induced upregulation of α-SMA (corresponding to Fig. 1H) protein expression (*p < 0.05, **p < 0.01 vs TGF-β2 treated alone group; n = 3; error bars represent SEM).


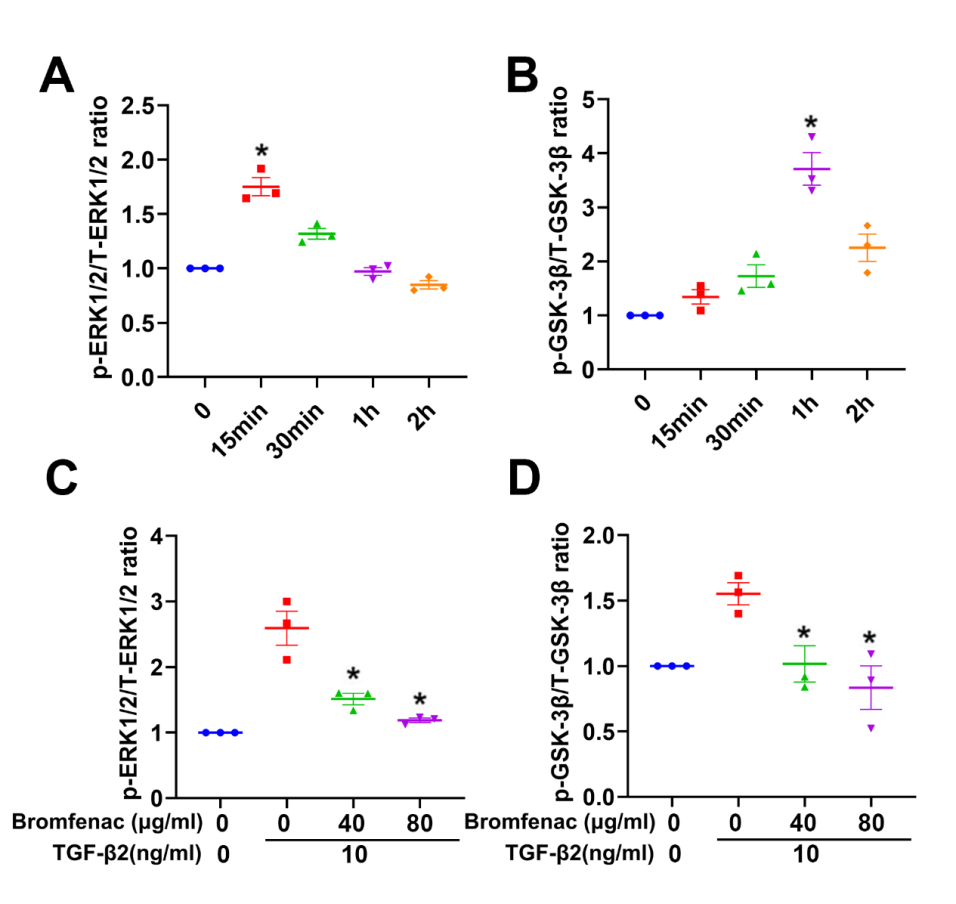


**Figure S2.** Quantification of p-ERK1/2 and p-GSK-3β protein levels by western blotting in HLEC-B3: (A) the protein expression of p-ERK1/2 (corresponding to Fig. 3A) stimulated by TGF-β2 for different time point detected; (B) the protein expression of p-GSK-3β (corresponding to Fig. 3A) stimulated by TGF-β2 for different time point detected (*p < 0.05, **p < 0.01 vs TGF-β2 free group); (C) effects of bromfenac in different concentrations on the TGF-β2-induced upregulation of p-ERK1/2 (corresponding to Fig. 3B) expression; (D) effects of bromfenac in different concentrations on the TGF-β2-induced upregulation of p-GSK-3β (corresponding to Fig. 3B) expression (*p < 0.05, **p < 0.01 vs TGF-β2 treated alone group; n = 3; error bars represent SEM).


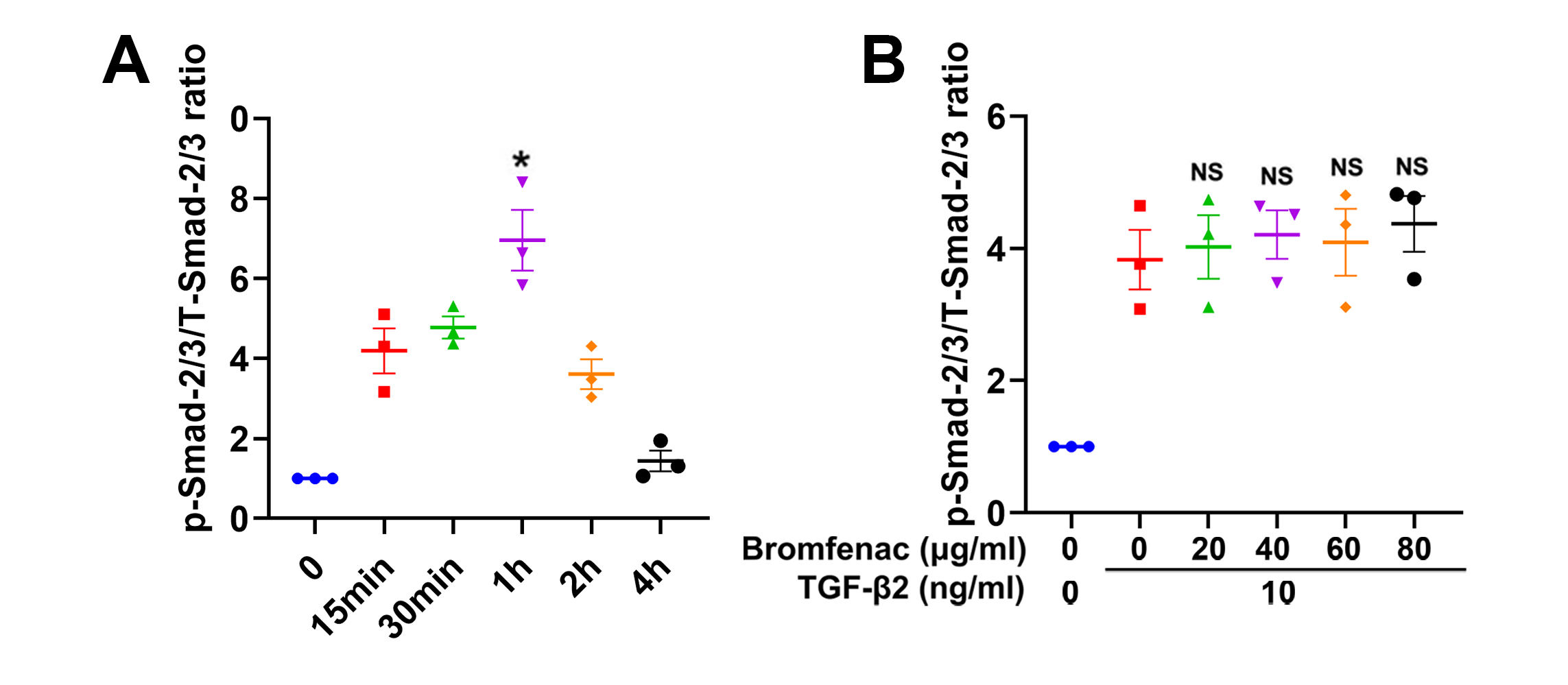


**Figure S3.** Quantification of p-smad2/3 protein levels by western blotting in HLEC-B3: (A) the protein expression of p-smad2/3 (corresponding to Fig. 3C) stimulated by TGF-β2 for different time point detected (**p < 0.01 vs TGF-β2 free group); (B) effects of bromfenac in different concentrations on the TGF-β2-induced upregulation of p-smad2/3 (corresponding to Fig. 3D) expression (NS represents no significance compared with the TGF-β2 treated alone group, n = 3, error bars represent SEM).


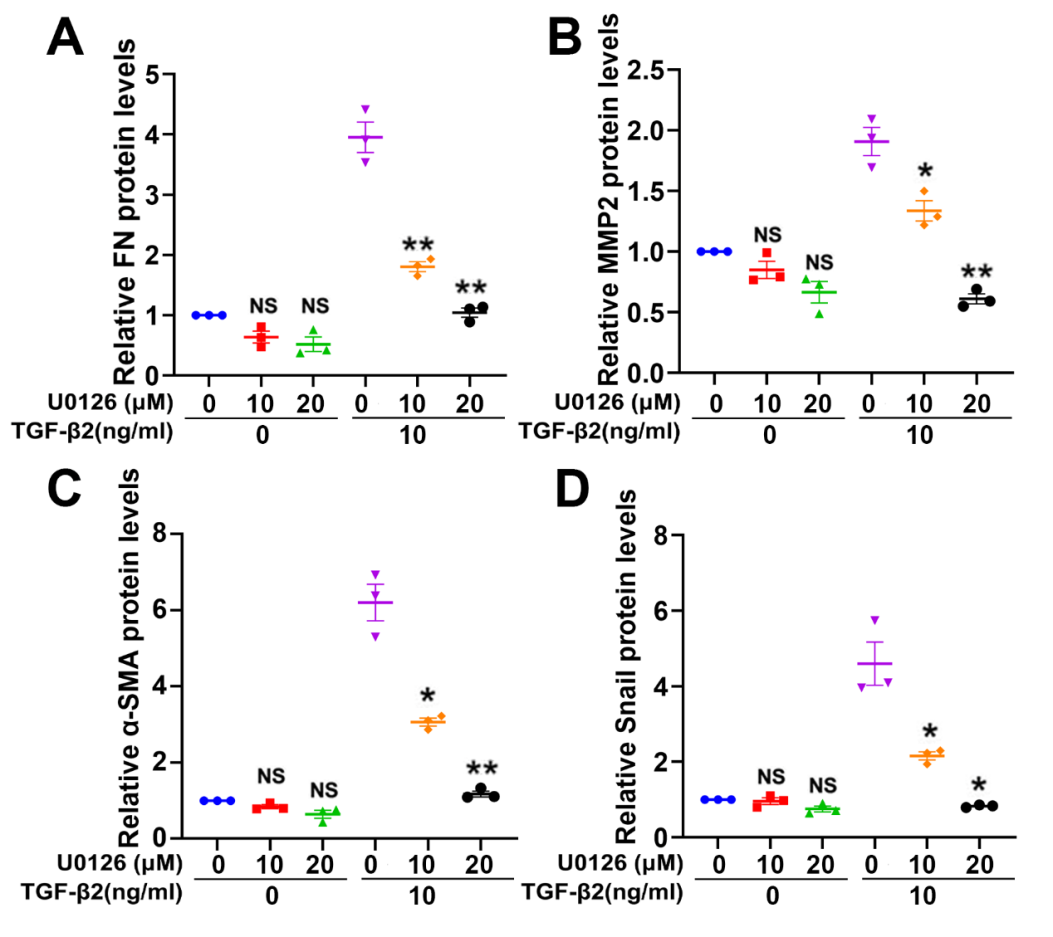


**Figure S4.** Quantification of FN, MMP2, α-SMA, and Snail protein levels by western blotting in HLEC-B3: pre-treatment with U0126 for 2 h suppressed the TGF-β2-induced upregulation of FN (A), MMP2 (B), α-SMA (C), and Snail (D) protein expression (corresponding to Fig. 3I, *p < 0.05, **p < 0.01 vs TGF-β2 treated alone group, NS represents no significance compared with the control group, n = 3, error bars represent SEM).


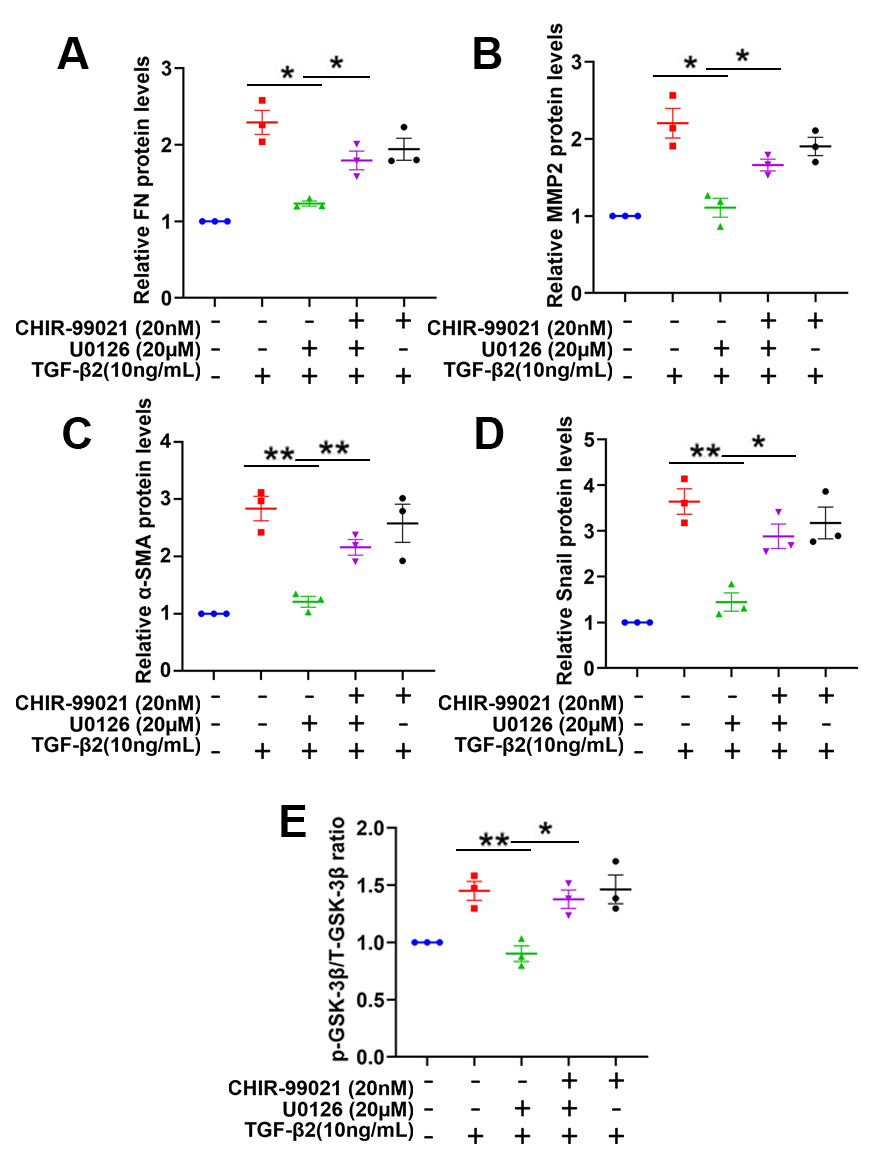


**Figure S5.** Quantification of FN, MMP2, α-SMA, Snail, and p-GSK-3β protein levels by western blotting in HLEC-B3 after treatment with CHIR-99021 and U0126: the cells were treated with CHIR-99021 for 24 h and U0126 for 2 h before TGF-β2 treatment. The protein expression of FN (A), MMP2 (B), α-SMA (C), Snail (D) and p-GSK-3β (E), respectively (corresponding to Fig. 4E and 4F, *p < 0.05, **p < 0.01 between groups, n = 3, error bars represent SEM).


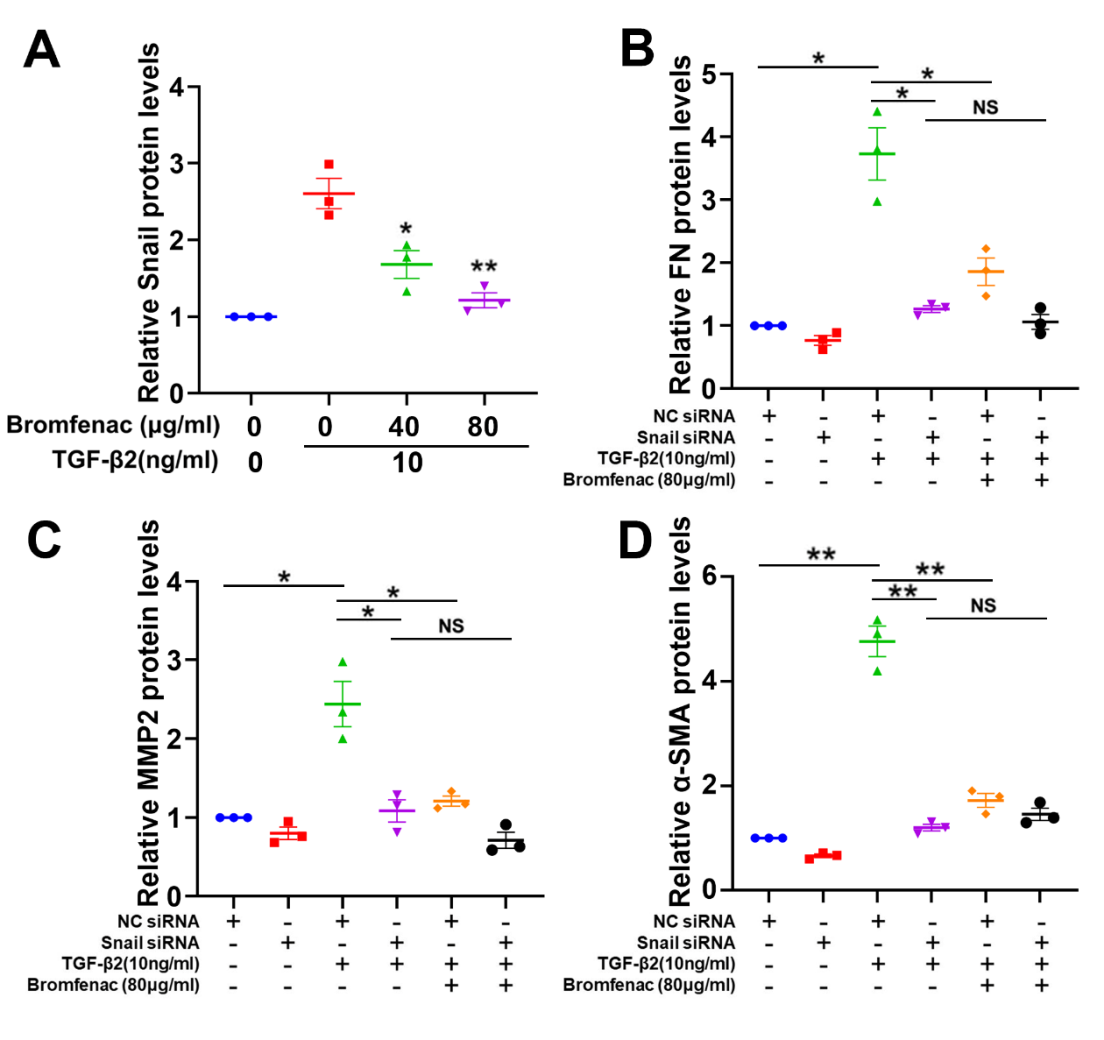


**Figure S6.** The role of Snail in TGF-β2-induced EMT: the cells were transfected with Snail siRNA or NC siRNA before bromfenac and TGF-β2 treatment. (A) Quantification of Snail (corresponding to Fig. 4G) protein levels by western blotting in HLEC-B3: effects of bromfenac in different concentrations on the TGF-β2-induced upregulation of Snail expression (*p < 0.05, **p < 0.01 vs TGF-β2 treated alone group). The quantification of protein expression of FN (B), MMP2 (C), and α-SMA (D) detected by western blot analysis, respectively (corresponding to Fig. 4H, *p < 0.05, **p < 0.01 between groups; n = 3; error bars represent SEM).


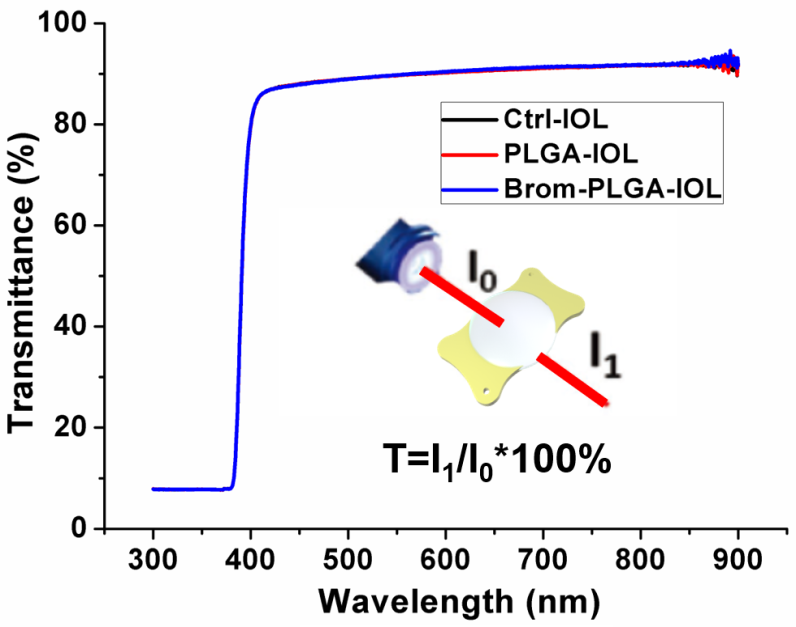


**Figure S7.** The transmittance of Ctrl-IOL, PLGA-IOL, and Brom-PLGA-IOL.


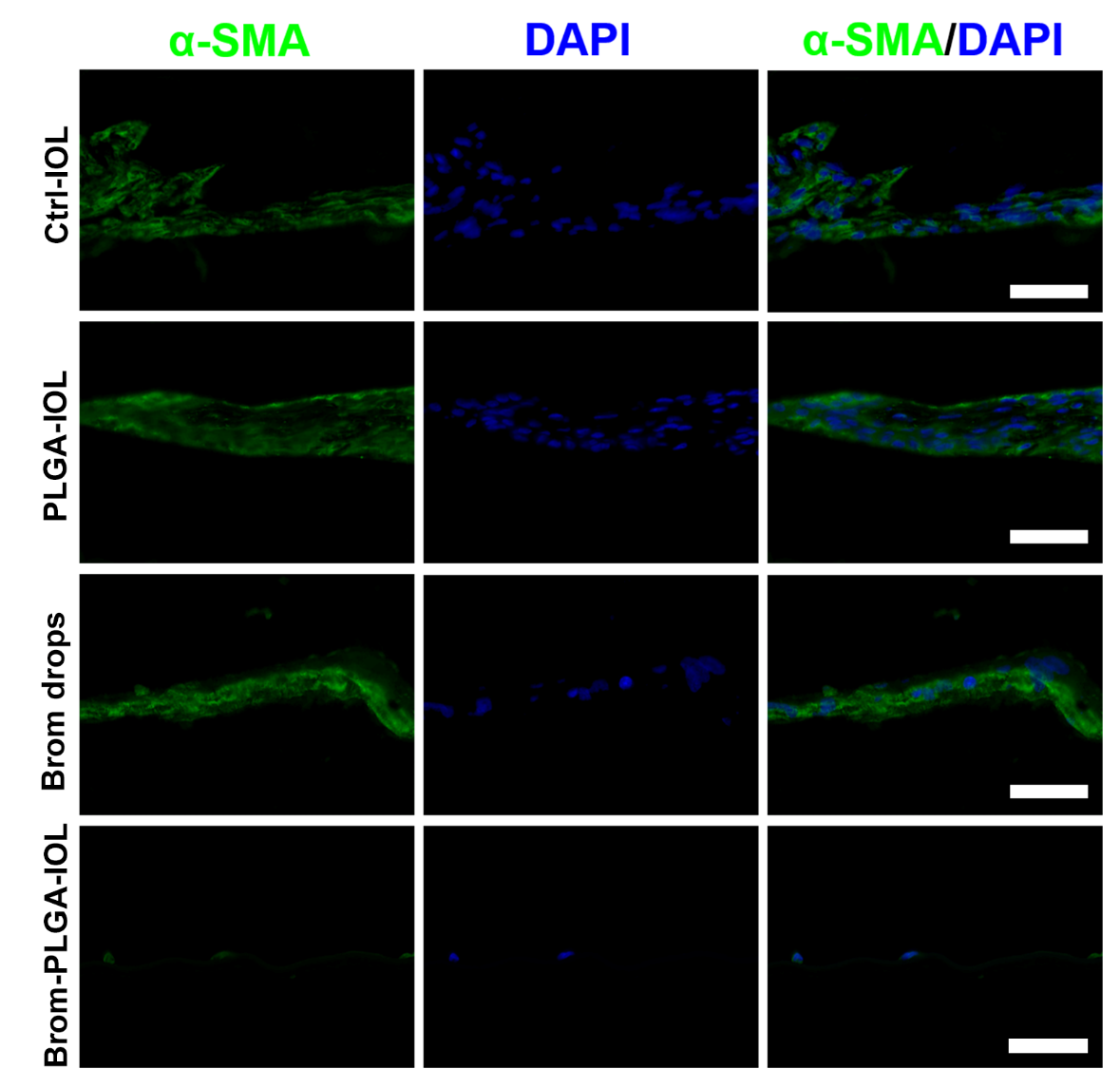


**Figure S8.** Immunofluorescence assay of posterior capsule stained with α-SMA (green). The results showed that the posterior capsules, with few cells, remained smooth in the bromfenac-PLGA-IOL group, while a large number of cells accumulated the posterior capsules in the other three groups (scale bar, 50 μm).
